# Change of genitourinary cancer patients’ perception and expectations over the course of pharmacotherapy

**DOI:** 10.1371/journal.pone.0278039

**Published:** 2022-11-22

**Authors:** Yoshiaki Satou, Kousuke Ieiri, Takahito Negishi, Nobuki Furubayashi, Motonobu Nakamura

**Affiliations:** Department of Urology, National Hospital Organization Kyushu Cancer Center, Fukuoka, Japan; IRCCS Giovanni Paolo II Cancer Hospital, ITALY

## Abstract

To determine the course of treatment while considering the patients’ desires, we examined trends regarding patients’ perception and expectations over the course of cancer pharmacotherapy. We retrospectively reviewed interview sheets filled in by patients with advanced urogenital cancers when they started a new pharmacotherapy regimen between 2014 and 2020. The responses to the following questions were analyzed: 1) How did your doctor explain the treatment objectives?; 2) Are you willing to receive treatment?; and 3) When the standard treatment becomes difficult to continue, would you like to try another treatment even if it may cause severe side effects? A total of 277 patients answered the interview sheet. The percentage of patients who accurately perceived the treatment objectives among patients receiving 1^st^, 2^nd^, and 3^rd^ line regimens was 67%, 79%, and 93%, respectively. The percentage significantly improved over the course of pharmacotherapy (p = 0.0057). The percentage of patients who indicated that they were willing to receive treatment in 1^st^, 2^nd^, and 3^rd^ line regimens was 80%, 83%, and 86%, respectively. The percentage of patients who indicated that they wanted to try another treatment when the standard treatment became difficult to continue in 1^st^, 2^nd^, and 3^rd^ line regimens was 56%, 64%, and 59%, respectively. The percentage of patients who accurately perceived the objective of pharmacotherapy increased over the course of pharmacotherapy. The rate of patients who were willing to receive treatment and try other treatments when the standard treatment became too difficult to continue remained consistently high.

## Introduction

Pharmacotherapy is the standard treatment for patients with metastatic or locally advanced cancers. It can prolong survival or improve symptoms; however, it is not usually curative and may cause adverse effects that can be life-threatening and which lead to the deterioration of the patient’s quality of life [1–3].

A previous study showed that chemotherapy does not always prolong survival and may worsen quality of life [4]. Aggressive cancer treatment is not always beneficial for patients; thus, it is important to make informed decisions about whether to receive chemotherapy in consideration of the patient’s perception and expectations.

Recently, in the field of urogenital cancer, new agents such as molecular targeted agents and immune checkpoint inhibitors have been developed [5–7]. Furthermore, numerous new treatment strategies are under development to improve the prognosis of advanced urogenital cancer [8–10]. These provide several treatment options and extend the treatment period [11,12]. Several studies have reported on patients’ perceptions at the introduction of the first pharmacotherapy [13,14]. However, the change in patients’ perceptions and expectations over the course of pharmacotherapy treatment are not well characterized.

In our institution, every time we start a new regimen, we confirm the patient’s perception and expectations using a questionnaire for advanced care planning. To determine the course of treatment while considering the patients’ desires, we examined the trends in patients’ perception and expectations over the course of cancer pharmacotherapy.

## Patients and methods

### Patients

Genitourinary cancer patients with metastases or locally advanced diseases were admitted to hospital at the time of the introduction of new pharmacotherapy regimens. We obtained their informed consent, information about disease condition, and a treatment overview in the outpatient clinic or during admission. Each time we start a new pharmacotherapy regimen, we confirm the patient’s perception and expectations about treatment using an interview sheet for advanced care planning. We retrospectively reviewed the interview sheets completed by patients who underwent 1^st^ line to 3^rd^ line pharmacotherapy at our institution between June 2014 and March 2020. This study was approved by the institutional review board of National Hospital Organization Kyushu Cancer Center (approval no. 2020–4) and respective institutions. Additional informed consent from patients was not required by the Institutional Review Board of National Hospital Organization Kyushu Cancer Center for this retrospective study.

### Questionnaire

We extracted and analyzed three questions from the interview sheet. Question-1 was “How did your doctor explain the treatment objectives?”. The response options were “complete cure”, “shrink the tumor”, “improve symptoms”, “don’t know” and “not explained”. Question 2 was “Are you willing to receive the treatment?”. The response options were “very likely”, “somewhat likely”, “not so much”, “not at all” and “don’t know”. Question 3 was “When the standard treatment becomes difficult to continue, would you like to try another treatment for which there is a slight chance of response, even if it may cause severe side effects?”. The response options were “very likely”, “somewhat likely”, “not so much”, “not at all” and “don’t know”. Non-responses were also recorded.

### Statistical analysis

For the statistical analysis, we divided the responses into two groups as follows. With regard to Question 1, “shrink the tumor” and “improve symptoms” were classified as responses that reflected an accurate perception, while “complete cure”, “don’t know” and “not explained” were classified as responses that reflected an inaccurate perception. With regard to Questions 2 and 3, “very likely” and “somewhat likely” were classified as responses that indicated willingness, while “not so much” and “not at all” were classified as responses that indicated reluctance. The change in the response rate over the course of pharmacotherapy was analyzed using Pearson’s chi-squared test. Factors associated with accurate perception in Question 1 and willingness in Questions 2 and 3 were investigated in a multivariable logistic regression analysis. All analyses were conducted with the exclusion of non-responses. Factors associated with willingness in Questions 2 and 3 were calculated with the exclusion of “don’t know”. Two-sided p values of <0.05 were considered to indicate statistical significance. All analyses were performed using the JMP^®^ Pro software package (version 15.1.0, SAS Institute, Inc., Cary, NC, USA).

## Results

### Patient characteristics

The patient characteristics are presented in Table 1. A total of 277 patients answered the interview sheet (1st line chemotherapy, n = 145; 2nd line chemotherapy, n = 95; 3rd line chemotherapy, n = 37). The median age at the initiation of the 1^st^ line, 2^nd^ line, and 3^rd^ line regimen was 67, 70, and 67 years, respectively. The total numbers of patients with renal cell carcinoma, urothelial carcinoma, and prostate cancer were 95, 116, and 66, respectively. The total numbers of patients who received cytotoxic chemotherapy, targeted therapy, and immune-checkpoint inhibitor therapy were 151, 56, and 70, respectively.

**Table 1:** Patient characteristics.

		Total		1st line		2nd line		3rd line	
		(n = 277)		(n = 145)		(n = 95)		(n = 37)	
Age at the initiation of chemotherapy		70	(26–88)	67	(26–84)	70	(44–88)	67	(43–84)
Sex									
	Male	208	(75)	113	(78)	73	(77)	22	(59)
	Female	69	(25)	32	(32)	22	(23)	15	(41)
Cancer location									
	Kidney	95	(34)	53	(37)	27	(29)	15	(41)
	Bladder/Upper urinary tract	116	(42)	52	(36)	42	(44)	22	(59)
	Prostate	66	(24)	40	(27)	26	(28)	0	
Treatment agent									
	Cytotoxic chemotherapy	151	(55)	90	(62)	46	(48)	15	(41)
	Targeted therapy	56	(20)	37	(26)	11	(12)	8	(22)
	Immune-checkpoint inhibitor	70	(25)	18	(12)	38	(40)	14	(37)

Data are presented as the median (range) or a n (%).

### Patients’ perception of chemotherapy

**Fig 1:**
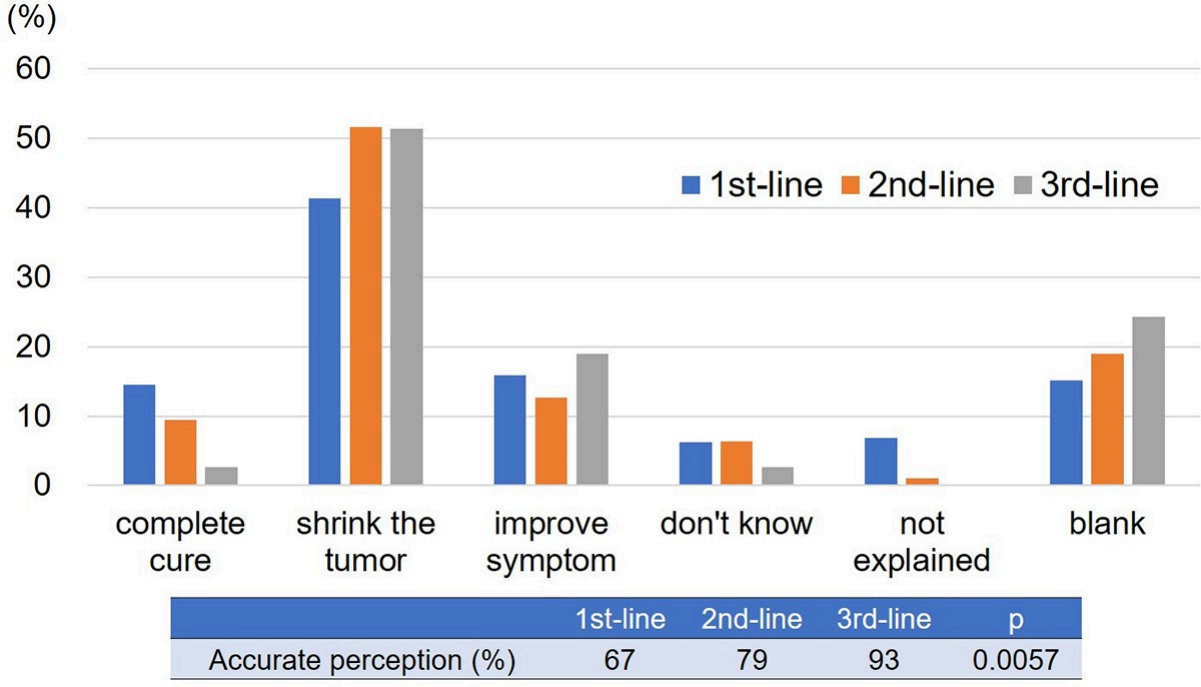
The response and the change in the response rate to Question 1. The percentage of patients who accurately perceived the objective of pharmacotherapy increased over the course of pharmacotherapy.

The responses to Question 1, “How did your doctor the objectives?” in each treatment line are shown in Fig 1. The percentage of patients who accurately perceived the treatment objectives among patients receiving 1^st^, 2^nd^, and 3^rd^ line regimens was 67%, 79%, and 93%, respectively. This significantly improved over the course of pharmacotherapy (p = 0.0057). A multivariable logistic regression analysis also demonstrated a significant association between the treatment line and accurate perception (S1 Table).

### Willingness to receive treatment

**Fig 2:**
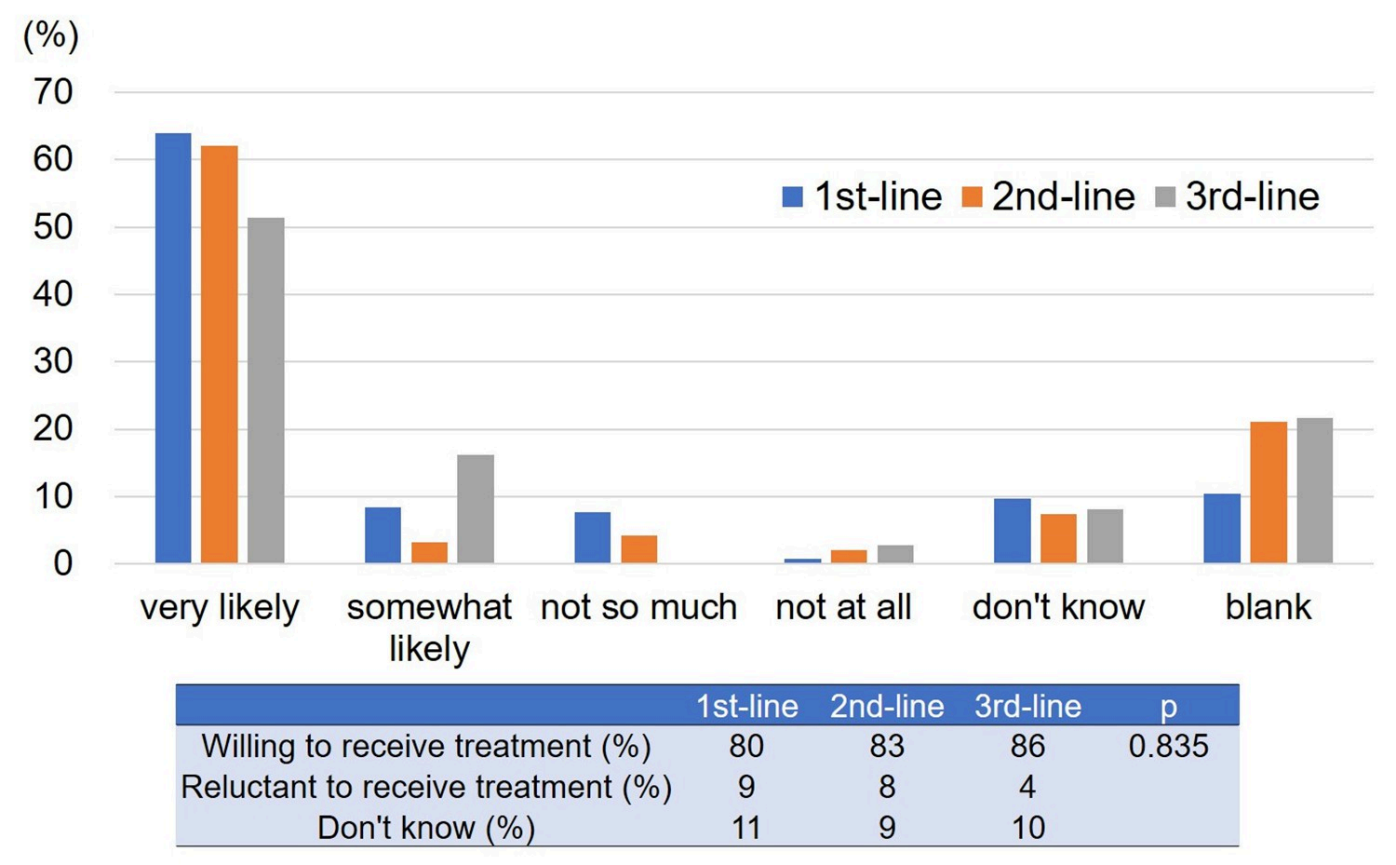
The response and the change in the response rate to Question 2. The patients who were indicated that they were willing to receive treatment remained >80% in the 1^st^ to 3^rd^ treatment line.

The responses to Question 2, “Are you willing to receive the treatment?” in each treatment line are shown in Fig 2. The patients who were indicated that they were willing to receive treatment remained >80% in the 1^st^ to 3^rd^ treatment line, whereas patients who were reluctant to receive treatment were <10% in each treatment line. We did not identify any factors associated with willingness to receive treatment (S2 Table).

### Willingness to try another treatment when standard treatment becomes difficult to continue

**Fig 3:**
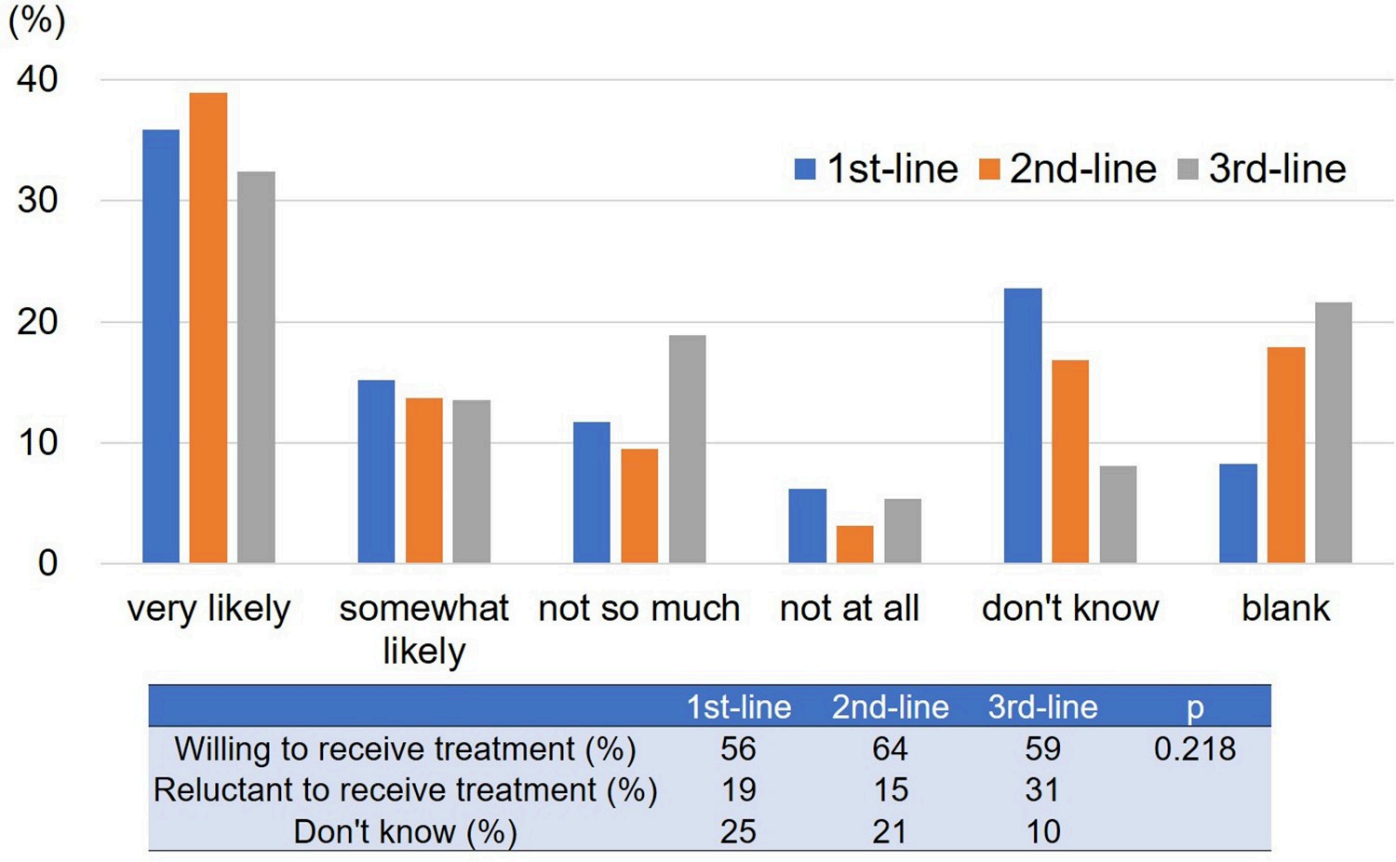
The response and the change in the response rate to Question 3. Approximately 60% of patients responded positively in 1^st^ to 3^rd^ treatment line.

The responses to the Question 3 “When the standard treatment becomes difficult to continue, would you like to try another treatment, even if it may cause severe side effects?” in each treatment line are shown in Fig 3. Approximately 60% of patients responded positively in 1^st^ to 3^rd^ treatment line. On the other hand, the rate of “don’t know” decreased with the treatment line, although this decrease was not statistically significant. We did not identify any factors that were significantly associated with willingness (S3 Table).

## Discussion

Pharmacotherapy can prolong the prognosis of patients with advanced urogenital cancer, however in most of these cases, curative treatment is not possible [5–7]. Therefore, it is important for patients to accurately understand the objective of pharmacotherapy. In previous reports, the rate of accurate perception at the beginning of the 1^st^ line regimen ranged from 33% to 68% [13,15,16]. In our study 67% of the patients accurately perceived the objective of pharmacotherapy at the initiation of the 1^st^ line therapy regimen. The rate of accurate perception was relatively higher in comparison to previous reports, because the study was conducted in a single cancer center and the patients were informed of their disease condition not only by physicians but also by nurses.

The proportion of patients who accurately perceived the treatment objectives significantly increased from the 1^st^ line to the 3^rd^ line, as follows: 1^st^ line, 67%; 2^nd^ line, 79%; and 3^rd^ line, 93%, which indicates that the perception of pharmacotherapy among the patients was correctly modified with the course of pharmacotherapy. Previous studies reported that the rate of correct understanding improved over time [15,16]. It supported our findings and indicated that sequential therapy with the passage of helped to correct patient perceptions. On the other hand, at the beginning of treatment, some patients incorrectly perceived the objective of pharmacotherapy and support from medical staff should be considered.

More than 80% of patients were willing to receive treatment and the proportion in 3^rd^ line therapy tended to increase in comparison to 1^st^ line therapy. Maintaining hope has been reported to be essential for patients with advanced cancer [17,18]. Continuing pharmacotherapy may provide hope for patients; thus, the majority of patients were willing to receive treatment, even in late line therapy.

Approximately 60% of patients answered that they were willing to try another treatment when the standard treatment becomes difficult to continue. There was little difference in the proportions among the treatment lines. In general, experimental treatments, for which there is not a high expectation of response, and which can cause severe toxicity, are considered after standard treatment. Despite this situation, the majority of patients were willing to try non-standard treatment because the small chance of therapeutic benefit could be regarded as reasonable justification for receiving non-standard treatment, and because patients tended to discount potential toxicity [17,19]. Physicians should follow patient requirements as much as possible; however, it should be kept in mind that palliative chemotherapy for progressive metastatic cancer can worsen the quality of life near death [4]. For this reason, the indications for non-standard treatment should be determined with cautious deliberation.

The present study was associated with some limitations. The present study was conducted in a regional cancer center where there was stronger support and earlier integration of palliative care for patients in comparison to general hospitals. This may have increased the number of patients who accurately perceived the objective of pharmacotherapy and led to overestimation of the rate of correct understanding [20]. Furthermore, the cohort of the study was heterogeneous, containing patients with prostate, kidney, and urothelial cancer who were treated with cytotoxic chemotherapy, targeted therapy, and immune-checkpoint inhibitors. The side effects or treatment efficacy, which can influence the results, were not considered in the analysis. However, all of the patients in the cohort had been diagnosed with advanced urogenital cancer and their prognosis was generally a few years [7,21,22]. Therefore, we believe that the present study provided information related to chemotherapy with non-curative intent.

The present study showed that there may be discrepancy in the perception of non-curative pharmacotherapy between patients and physicians, and that patients are willing to continue treatment beyond the 1^st^ line therapy. Patient involvement in decision-making for cancer treatment has been shown to improve patients’ quality of life, physical functioning, and patient satisfaction [23]. Although it is not always easy to select appropriate treatment options in the non-curative setting, in order to continue pharmacotherapy for the patient’s benefit, it is important for physicians to share the treatment goal and grasp the patient’s desire.

## Conclusion

The percentage of patients who accurately perceived the objective of pharmacotherapy increased over the course of pharmacotherapy. Beyond 1^st^ line therapy the percentage of patients who were willing to receive treatment remained high, and more than half of the patients indicated that they would be willing to try another treatment when the standard treatment becomes difficult to continue.

## Supporting information

S1 Table Multivariable logistic regression analyses to evaluate factors associated with accurate perception in Question 1. (DOCX)

S2 Table Multivariable logistic regression analyses to evaluate factors associated with willingness in Questions 2. (DOCX)

S3 Table Multivariable logistic regression analyses to evaluate factors associated with willingness in Questions 3. (DOCX)
