# Supplementary material for: Change of genitourinary cancer patients’ perception and expectations over the course of pharmacotherapy
Source: PLoS One. 2022 Nov 22;17(11):e0278039. doi: 10.1371/journal.pone.0278039 (PMC9681061; doi:10.1371/journal.pone.0278039)
Supplement: S1 Table — (DOCX) [file pone.0278039.s001.docx]

|  |  |  | Univariate Analysis | | | Multivariate Analysis | | |
| --- | --- | --- | --- | --- | --- | --- | --- | --- |
|  |  | n | HR (95%CI) | | p | HR (95%CI) | | p |
| Gender | |  |  |  |  |  |  |  |
|  | Male | 172 | 1 | (reference) |  | 1 | (reference) |  |
|  | Female | 56 | 1.17 | (0.58-2.38) | 0.660 | 1.28 | (0.58-2.80) | 0.543 |
| Age at the initiation of the regimen | |  |  |  |  |  |  |  |
|  | ≤74 | 164 | 1 | (reference) |  | 1 | (reference) |  |
|  | ≥75 | 64 | 0.74 | (0.39-1.41) | 0.358 | 0.823 | (0.40-1.69) | 0.597 |
| Performance status | |  |  |  |  |  |  |  |
|  | 0/1 | 201 | 1 | (reference) |  | 1 | (reference) |  |
|  | 2/3 | 20 | 1.05 | (0.36-3.02) | 0.932 | 1.38 | (0.43-4.36) | 0.588 |
|  | unknown | 7 |  |  |  |  |  |  |
| Types of cancer | |  |  |  |  |  |  |  |
|  | Prostate cancer | 54 | 1 | (reference) |  | 1 | (reference) |  |
|  | Kidney cancer | 79 | 0.49 | (0.21-1.16) | 0.103 | 1.13 | (0.22-5.80) | 0.878 |
|  | Urothelial cancer | 95 | 0.53 | (0.23-1.24) | 0.142 | 0.43 | (0.16-1.13) | 0.086 |
| Pharmacotherapy agent | |  |  |  |  |  |  |  |
|  | Cytotoxic chemotherapy | 122 | 1 | (reference) |  | 1 | (reference) |  |
|  | Targeted therapy | 48 | 0.24 | (0.052-1.09) | 0.066 | 0.24 | (0.051-1.11) | 0.067 |
|  | Immune checkpoint inhibitor | 58 | 0.42 | (0.14-1.27) | 0.124 | 0.42 | (0.14-1.29) | 0.130 |
| Treatment line | |  |  |  |  |  |  |  |
|  | 1st line | 123 | 1 | (reference) |  | 1 | (reference) |  |
|  | 2nd line | 77 | 1.84 | (0.94-3.58) | 0.074 | 2.44 | (1.10-5.41) | 0.028 |
|  | 3rd line | 28 | 6.27 | (1.42-27.71) | 0.016 | 8.23 | (1.74-38.96) | 0.008 |
