# Supplementary material for: Change of genitourinary cancer patients’ perception and expectations over the course of pharmacotherapy
Source: PLoS One. 2022 Nov 22;17(11):e0278039. doi: 10.1371/journal.pone.0278039 (PMC9681061; doi:10.1371/journal.pone.0278039)
Supplement: S2 Table — (DOCX) [file pone.0278039.s002.docx]

|  |  |  | Univariate Analysis | | | Multivariate Analysis | | |
| --- | --- | --- | --- | --- | --- | --- | --- | --- |
|  |  | n | HR (95%CI) | | p | HR (95%CI) | | p |
| Gender |  |  |  |  |  |  |  |  |
|  | Male | 154 | 1 | (reference) |  | 1 | (reference) |  |
|  | Female | 55 | 7.14 | (0.93-54.87) | 0.059 | 7.23 | (0.89-58.55) | 0.064 |
| Age at the initiation of the regimen | |  |  |  |  |  |  |  |
|  | ≤74 | 150 | 1 | (reference) |  | 1 | (reference) |  |
|  | ≥75 | 59 | 1.19 | (0.43-3.30) | 0.734 | 1.00 | (0.35-2.89) | 0.995 |
| Performance status | |  |  |  |  |  |  |  |
|  | 0/1 | 188 | 1 | (reference) |  | 1 | (reference) |  |
|  | 2/3 | 16 | 0.40 | (0.10-1.56) | 0.189 | 0.40 | (0.094-1.73) | 0.221 |
|  | unknown | 5 |  |  |  |  |  |  |
| Types of cancer | |  |  |  |  |  |  |  |
|  | Prostate cancer | 51 | 1 | (reference) |  | 1 | (reference) |  |
|  | Kidney cancer | 70 | 1.20 | (0.38-3.81) | 0.757 | 0.28 | (0.023-3.40) | 0.318 |
|  | Urothelial cancer | 88 | 1.82 | (0.56-5.98) | 0.322 | 1.04 | (0.28-3.83) | 0.953 |
| Pharmacotherapy agent | |  |  |  |  |  |  |  |
|  | Cytotoxic chemotherapy | 114 | 1 | (reference) |  | 1 | (reference) |  |
|  | Targeted therapy | 43 | 1.04 | (0.31-3.46) | 0.947 | 3.38 | (0.26-43.86) | 0.352 |
|  | Immune checkpoint inhibitor | 52 | 1.28 | (0.39-4.23) | 0.684 | 2.16 | (0.28-16.67) | 0.459 |
| Treatment line | |  |  |  |  |  |  |  |
|  | 1st line | 115 | 1 | (reference) |  | 1 | (reference) |  |
|  | 2nd line | 68 | 1.2 | (0.43-3.37) | 0.724 | 1.01 | (0.33-3.09) | 0.989 |
|  | 3rd line | 26 | 2.91 | (0.36-23.46) | 0.315 | 2.39 | (0.26-21.71) | 0.439 |

(B)
