# Supplementary material for: Change of genitourinary cancer patients’ perception and expectations over the course of pharmacotherapy
Source: PLoS One. 2022 Nov 22;17(11):e0278039. doi: 10.1371/journal.pone.0278039 (PMC9681061; doi:10.1371/journal.pone.0278039)
Supplement: S3 Table — (DOCX) [file pone.0278039.s003.docx]

|  |  |  | Univariate Analysis | | | Multivariate Analysis | | |
| --- | --- | --- | --- | --- | --- | --- | --- | --- |
|  |  | n | HR (95%CI) | | p | HR (95%CI) | | p |
| Gender |  |  |  |  |  |  |  |  |
|  | Male | 140 | 1 | (reference) |  | 1 | (reference) |  |
|  | Female | 48 | 0.75 | (0.36-1.56) | 0.441 | 0.74 | (0.32-1.68) | 0.469 |
| Age at the initiation of the regimen | |  |  |  |  |  |  |  |
|  | ≤74 | 137 | 1 | (reference) |  | 1 | (reference) |  |
|  | ≥75 | 51 | 0.49 | (0.24-1.00) | 0.049 | 0.54 | (0.25-1.19) | 0.128 |
| Performance status | |  |  |  |  |  |  |  |
|  | 0/1 | 168 | 1 | (reference) |  | 1 | (reference) |  |
|  | 2/3 | 14 | 0.56 | (0.18-1.78) | 0.327 | 0.58 | (0.17-1.91) | 0.367 |
|  | unknown | 6 |  |  |  |  |  |  |
| Types of cancer | |  |  |  |  |  |  |  |
|  | Prostate cancer | 47 | 1 | (reference) |  | 1 | (reference) |  |
|  | Kidney cancer | 58 | 0.33 | (0.13-0.88) | 0.0259 | 0.23 | (0.036-1.41) | 0.112 |
|  | Urothelial cancer | 83 | 0.55 | (0.21-1.42) | 0.218 | 0.64 | (0.22-1.86 | 0.408 |
| Pharmacotherapy agent | |  |  |  |  |  |  |  |
|  | Cytotoxic chemotherapy | 104 | 1 | (reference) |  | 1 | (reference) |  |
|  | Targeted therapy | 46 | 0.52 | (0.23-1.19) | 0.522 | 1.82 | (0.32-10.44) | 0.503 |
|  | Immune checkpoint inhibitor | 38 | 0.86 | (0.38-1.97) | 0.864 | 1.82 | (0.49-6.71) | 0.370 |
| Treatment line | |  |  |  |  |  |  |  |
|  | 1st line | 100 | 1 | (reference) |  | 1 | (reference) |  |
|  | 2nd line | 62 | 1.46 | (0.68-3.17) | 0.334 | 1.40 | (0.59-3.29) | 0.443 |
|  | 3rd line | 26 | 0.66 | (0.26-1.67) | 0.664 | 0.87 | (0.30-2.53) | 0.791 |

(C)
